# Genome-wide identification of candidate chemosensory receptors in the bean bug *Riptortus pedestris* (Hemiptera: Alydidae) and the functional verification of its odorant receptor co-receptor (Orco) in recognizing aggregation pheromone

**DOI:** 10.3389/fphys.2023.1224009

**Published:** 2023-07-14

**Authors:** Panjing Liu, Jianglong Guo, Hongyi Wei, Likai Feng, Zhanlin Gao, Tao Zhang

**Affiliations:** ^1^ Key Laboratory of IPM on Crops in Northern Region of North China, Ministry of Agriculture, Institute of Plant Protection, Hebei Academy of Agriculture and Forestry Sciences, Integrated Pest Management Center of Hebei Province, Baoding, China; ^2^ Institute of Entomology, Jiangxi Agricultural University, Nanchang, China; ^3^ Institute of Plant Protection, Xinjiang Academy of Agricultural and Reclamation Sciences, Shihezi, China

**Keywords:** the bean bug, olfactory recognition, genome-wide, pheromone, RNAi

## Abstract

A sophisticated and sensitive olfactory system plays a vital role in the survival and reproduction of insects. Chemosensory receptors are indispensable for the molecular recognition and discrimination of semiochemicals. *Riptortus pedestris* is a notorious pest of legume plants, resulting in yield losses and quality decreases in soybeans. It is well accepted that *R. pedestris* highly relies on its olfactory system in detecting aggregation pheromones, host volatiles, and pesticides; however, little research focused on its chemosensory receptors. In the present study, we identified 237 odorant receptors (ORs), 42 gustatory receptors (GRs), and 31 ionotropic receptors (IRs) from the reported genome of *R. pedestris*, and analyzed their phylogenetic relationship with other hemipteran species. Through the results of RNA-seq and real-time quantitative PCR (qRT-PCR), we found that RpedORs displayed different expression levels in the antennae of *R. pedestris* at different development stages. To further verify the function of odorant receptor co-receptor (Orco), an obligate and unique insect OR, we silenced *RpedOrco* by RNA interference (RNAi) method. The results showed that silencing *RpedOrco* could significantly impair the response to aggregation pheromone in *R. pedestris*, indicating that *RpedOrco* plays an essential role in odorant detection. Our results can provide the theoretical foundations for revealing the olfactory recognition mechanism of *R. pedestris* and help explore and develop novel olfactory-based agents against this pest.

## 1 Introduction

Insects rely on their accurate olfactory systems to recognize chemical signals such as pheromones and plant volatiles, and thereby adapt to different environments and ecological niches (Gadenne et al., 2016; Haverkamp et al., 2018). The recognition of chemical cues in insects is a spectacularly complex process. When hydrophobic odorant or taste molecules diffuse into insect sensory lymph through epidermal pores, they are rapidly recognized, bound, and dissolved by odorant binding proteins (OBPs) or chemical sensory proteins (CSPs). Subsequently, they are transported to the lumen cilia of olfactory receptor neurons (ORNs), where chemical signals are converted into electrical signals (Leal, 2013), then these signals are transferred to the central nervous system, which manipulates insects to make corresponding responses. In the process of converting semiochemicals into electrical signals in the peripheral nerve system, at least three main chemosensory receptor families are involved, including odorant receptors (ORs), gustatory receptors (GRs), and ionotropic receptors (IRs) (Fleischer et al., 2018; Wicher, 2018). At the terminal of odorant recognition, odorant signals are inactivated or degraded by various odorant degrading enzymes (ODEs) (Younus et al., 2017; Wang et al., 2021).

Insect ORs, first reported in the *Drosophila* genome (Gao et al., 1999; Vosshall et al., 1999), are seven transmembrane domain (7-TMD) proteins consisting of 350–500 amino acids (Robertson, 2019). ORs are mainly expressed in the dendritic membranes of the ORNs, and there are two classifications: odorant receptor co-receptor (Orco) and odorant-specific olfactory receptor proteins (ORx) (Smart et al., 2008; Tian et al., 2022). Orco (formerly designated as OR83b) has a highly-conserved sequence, while common ORs, even in sibling species are incredibly variable (Wicher and Miazzi, 2021). For instance, the OR members in the fly are extremely divergent, with an average amino acid identity of ≈20% (Wetzel et al., 2001). Insect ORs recognize odorant molecules through a special heterodimer composed of an ORx and an Orco. Heterodimer ORx-Orco forms an ion channel that allows cations to pass through when it binds specific odorant molecules (Sato et al., 2008). In this complex, ORx is responsible for the specificity, while Orco is an obligate factor for the localization, stability, and protein folding of each ORx (Stengl and Funk, 2013). If *Orco* is knocked out, the olfactory-related behavior of an insect would be altered because of the disruption or even abolition of its OR functional repertoire (Fan et al., 2022). The essential function of Orco has been validated in numerous insects through RNA interference (RNAi) or gene editing technology. For example, RNAi-based silencing of *Orco* in the hemimetabolous blood-sucking insect *Rhodnius prolixus* results in losing the ability to find hosts, reducing the number of eggs laid and decreasing the survival rate (Franco et al., 2016). Moreover, mutations of *Bombyx mori*, whose *Orco* gene was knocked out by CRISPR-Cas9, are influenced on the aspects of their larval feeding and adult mating behavior (Liu et al., 2017).

Like ORs, insect GRs also contain 300–500 amino acids. However, GR family members are usually abundant in the taste organs and play key roles in sensing carbon dioxide, sugar, bitter compounds, and taste pheromones (Agnihotri et al., 2016; Fleischer et al., 2018). Insect IRs are related to the ionotropic glutamate receptors (iGluRs) family. They have been identified in both olfactory and gustatory organs and reported to be responsible for detecting acids, aromatics, and nitrogen-containing compounds (Agnihotri et al., 2016; Wicher and Miazzi, 2021; Dong et al., 2023). Several GRs and IRs are expressed in the antennae and have been characterized to mediate multiple olfactory-related capabilities, such as pheromone detection (Jones et al., 2007; Benton et al., 2009; Croset et al., 2010; Koh et al., 2014). GRs, IRs, and ORs together form a complex olfactory reception system and participate in olfactory responses.

The bean bug, *Riptortus pedestris* (Fabricius) (Hemiptera: Alydidae), is a polyphagous pest attacking legume plants in many East Asian countries (e.g., China, Japan, South Korea, and Thailand) (Mizutani et al., 2011; Lim, 2013). In the past 2 decades, this pest has become the dominant pest in soybean fields (Do et al., 2014; Li et al., 2019; Zhang et al., 2022). Both adults and nymphs of *R. pedestris* absorb soybean nutrients through the piercing-sucking mouthparts, and result in soybean stay-green, a phenomenon of a lack of leaf senescence, pod abortion, and abnormal seeds (Hill et al., 2006; Sakuraba et al., 2015; Li et al., 2019). Outbreak of *R. pedestris* could cause soybean yield losses, quality decrease, and potential germination decline of seeds (Rahman and Lim, 2017; Ahn et al., 2019; Park et al., 2023). It is convinced that *R. pedestris* population highly relies on chemicals cues in their aggregation, location of hosts, and avoidance of adverse environment (Xu et al., 2021). *R. pedestris* individuals of all developmental stages and sexes are reported to be attracted by the intraspecific aggregation pheromones, which were identified as a 1:5:1 mixture of (*E*)-2-hexenyl (*Z*)-3-hexenoate (E2HZ3H), (*E*)-2-hexenyl (*E*)-2-hexenoate (E2HE2H) and myristyl isobutyrate (MI) (Leal et al., 1995; Rahman et al., 2018). Meanwhile, *R. pedestris* can recognize soybean through a particular blend of plant volatiles, including (*Z*)-3-hexenol, (*Z)*-3-hexenyl acetate, 4-ethylbenzaldehyde, α-farnesene, and methyl salicylate (Song et al., 2022). The bean bugs also show the ability to distinguish hosts treated with chemical insecticides, especially bifenthrin (Maharjan and Jung, 2015). These odorants are recognized through its complicated and sophisticated olfactory system, with various olfactory-related proteins involved. A previous report claimed that 188 ORs, 6 GRs, and two IRs were identified from the antennal transcriptome of *R. pedestris* (Song et al., 2017), but the sequence information remains undisclosed and unavailable. More recently, 49 OBPs and 25CSPs were annotated from *R. pedestris* genome data (Li et al., 2022), which is significantly more than the transcriptome-based identification (eight OBPs and eleven CSPs) (Song et al., 2017). The results trigger a prediction that more chemosensory receptors would be explored from the *R. pedestris* genome.

In the present study, we aimed to systematically identify chemosensory receptors from the genome of *R. pedestris*, explore their phylogenetic relationship with other Hemiptera insects, and determine their expression in the antennae of different development stages through RNA-seq and real-time quantitative PCR (qRT-PCR). We also verified the importance of *RpedOrco* in responding to aggregation pheromones using RNAi-based technology. Our results will provide a theoretical basis for further understanding of the olfactory recognition of *R. pedestris*.

## 2 Materials and methods

### 2.1 Identification and bioinformatics analysis of candidate chemosensory receptors

To identify candidate ORs, we used the OR sequences of *Adelphocoris lineolatus*, *Apolygus lucorum*, *Halyomorpha halys*, *T. elegans*, and *Yemma signatus* as template sequences to homologous blast with the *R. pedestris* genome database (https://ngdc.cncb.ac.cn/gwh/Assembly/18849/show) (An et al., 2016; Sun D. et al., 2020; Huang et al., 2021). As for GRs and IRs, the sequences of *A*. *lineolatus*, *Ap. lucorum*, *H. halys*, and *T. elegans* were selected as queries with an E-value of 1e^−5^ (He et al., 2020; Chen et al., 2021). Then, OR, GR, and IR genes were further verified by blasting against the NCBI non-redundant (Nr) database and removing genes of low identity (<30%). The candidate chemoreceptor genes were then validated by the Pfam protein family database (http://pfam.xfam.org/search) and InterProScan 5 (Jones et al., 2014).

### 2.2 Sequence analysis and phylogenetic tree construction

The chromosomal location data of chemosensory receptor genes were obtained from the *R. pedestris* GFF files and mapped onto the chromosomes using Mapchart 2.32. The transmembrane domains were predicted using TMHMM Server v.2.0 (https://services.healthtech.dtu.dk/service.php?TMHMM-2.0), and the graphical representation of RpedOrco was generated with TMRPres2D (http://bioinformatics.biol.uoa.gr/TMRPres2D/) (Harrison et al., 2018). Protein sequence alignment was performed using ClustalX-2.1, and the results were presented by GeneDoc software (http://nrbsc.org/gfx/genedoc). The phylogenetic tree of chemosensory receptors from *R. pedestris* and homologous species was constructed using the neighbor-joining method. Trees with 1000-fold bootstrap replication were viewed and decorated using iTOL online tools (https://itol.embl.de/) (Letunic et al., 2021).

### 2.3 Insect rearing and sample collection

*R. pedestris* cultures were fed with green beans and maintained at a temperature of 24°C ± 2°C under a 14:10 photoperiod (L:D) and 70% ± 5% relative humidity (RH) (Fu et al., 2021). The antennae samples were collected from nymphs of 2nd, 3rd, 4th, and 5th instar, and 3-day-old adults (females and males). Each sample contains 80 pairs of antennae. Collected samples were immediately frozen in liquid nitrogen and stored at −80°C for standby.

### 2.4 RNA-Seq analysis

Total RNA was extracted using TRIzol reagent (TransGen, Beijing, China) following the manufacturer’s instructions. One microgram of high-quality RNA per sample was used to construct cDNA libraries. cDNA library preparation and transcriptomic sequencing were performed by Sangon Biotech (Shanghai, China) following the previously described protocol (Wen et al., 2020). The clean reads of six transcriptomes were uploaded to the Sequence Read Archive (SRA) database with the accession numbers SRR21820231-SRR21820236. Clean reads generated from transcriptome were used to map the *R. pedestris* genome (https://ngdc.cncb.ac.cn/gwh/Assembly/18849/show) using HISAT2 (Kim et al., 2019). The clean readings mapped to the reported genome were listed in Supplementary Table S1 Read summarization was used to obtain gene expression levels using featureCounts, while the trimmed mean of M-values (TMM) was used to normalize the counts (Robinson et al., 2010; Liao et al., 2014). Tests for pairwise differential expression were performed in the DESeq2 R package with *p* < 0.05 (Love et al., 2014; Tang et al., 2021). Based on log-transformed TMM values, the expression levels of the 177 OR genes (over 300 aa) in *R. pedestris* antennae at different stages were visualized by the heatmap using TBtools (version 1.098728) (Chen et al., 2020).

### 2.5 Expression analysis of *RpedORs*

To estimate the consistency between RNA-seq and qRT-PCR data, we randomly selected 16 *RpedOR* genes to determine their expression in antennae on an ABI QuantStudio6 Q6 Real-Time PCR System (Applied Biosystems, Foster City, CA, United States of America). The primers for *RpedORs* and reference genes were designed by Primer 6.0 (Supplementary Table S2). qRT-PCR reactions were performed in a 20 μL reaction system containing 10 μL TransStart Tips Green Mix (TransGen, Beijing, China), 0.5 μL of each primer (10 μM), 1 μL of sample cDNA, and 8 μL of sterilized H_2_O. Three independent biological and three technical replicates were conducted for each sample. The relative expression of RpedORs was analyzed using the 2^−*ΔΔ*CT^ method (Livak and Schmittgen, 2001).

### 2.6 RNAi of *RpedOrco* gene

Double-stranded RNA (dsRNA) was synthesized using the T7 Ribomax Express RNAi System (Promega, Madison, United States), based on the fragment of *RpedOrco* that beforehand amplified using specific primers with T7 RNA polymerase promoter (Supplementary Table S2). And then, the quality and concentration of dsRNA were determined by agarose gel electrophoresis and Nanodrop 2000 spectrophotometer (Thermo, Wilmington, DE, United States), respectively. A double-stranded green fluorescent protein (dsGFP) fragment amplified from the *GFP* gene (GenBank No. U50963) was used as the negative control. The newly emerged *R. pedestris* were separated and reared individually before dsRNA injection. For each bug, 2 μg of dsOrco or dsGFP in 2 μL water was injected into the head of the adults using a microsyringe (Ikeno et al., 2011). The antennae of dsRNA-injected bugs were collected at 1, 3, 5, and 7 days of post-injection to evaluate the silencing efficiency of *RpedOrco* using qRT-PCR. Three independent biological repeats were carried out.

### 2.7 Dual choice bioassay

Dual-choice bioassays were conducted in a two-choice cage (1800 mm × 600 mm × 600 mm) to evaluate the influence of silencing the *RpedOrco* gene on *R. pedestris*’s response to aggregation pheromone. A handful of green beans with and without an aggregation pheromone lure (purchased from Beijing Pherobio Technology Co., ltd. China) were placed at two sides of the cage (Supplementary Figure S1). The lure was a ternary mixture of E2HZ3H, E2HE2H and MI at a ratio of 1:5:1. Ten dsOrco- or dsGFP-injected *R. pedestris* (5–7 days post-injection) individuals were released at the center. The bugs were allowed to make behavioral choices in 3 h, and after that, their final position was checked (Song et al., 2022). Three biological repeats were conducted for each treatment.

### 2.8 Statistical analysis

The statistical analysis was performed by SPSS (version 22.0) and the R (version 4.0.5). The differences for qRT-PCR data among six samples were subjected to one-way analysis of variance (ANOVA) with Tukey’s multiple comparison test. The significant differences of *RpedOrco* mRNA levels after injection and the dual-choice bioassays were analyzed by Student's *t*-test. *p* < 0.05 was considered to represent statistically significant differences between samples. The data were expressed as mean +standard error. Finally, the results were displayed with GraphPad Prism 8 software.

## 3 Results

### 3.1 Identification of ORs, GRs, and IRs

A total of 237 candidate RpedORs, 42 RpedGRs, and 31 RpedIRs were identified from the *R. pedestris* genome (Supplementary Table S3). The amino acid (aa) number of RpedOR sequences ranged from 103 to 1,131. Among them, 177 RpedORs were over 300 aa in sequence length. RpedOR2 had the most extended sequence (1,131 aa), significantly differing from typical insect ORs, presumably due to abnormal sequence splicing. In addition, we also noticed that RpedORs had relatively low amino acid identities (30.13%–69.88%) with the homologous ORs in other species according to the BLASTx results of NCBI (Supplementary Table S3). Furthermore, except for a few RpedORs (e.g., RpedOR3, RpedOR13, and RpedOR16, etc.), the majority of RpedORs significantly matched the 7tm_6 (PF02949) or olfactory receptor (IPR004117) domain (Supplementary Table S3).

The sequences of RpedGRs ranged from 113 to 499 aa, of which 23 RpedGRs are more than 300 aa. The homology search of the GR sequences using BLASTx showed that most of the RpedGRs matched those of *H. halys*, and some RpedGRs matched those of *T. elegans*, such as RpedGR2, RpedGR7, RpedGR19-20, RpedGR25, RpedGR30, RpedGR36 and RpedGR39 (Supplementary Table S3). As for candidate RpedIRs, the sequences ranged from 126 to 1,226 aa. Similar to RpedGRs, RpedIRs were mainly matched to those of *H. halys*, and *T. elegans*, with amino acid identities ranging from 31.39% to 92.23% (Supplementary Table S3).

### 3.2 Phylogenetic analysis

**FIGURE 1:**
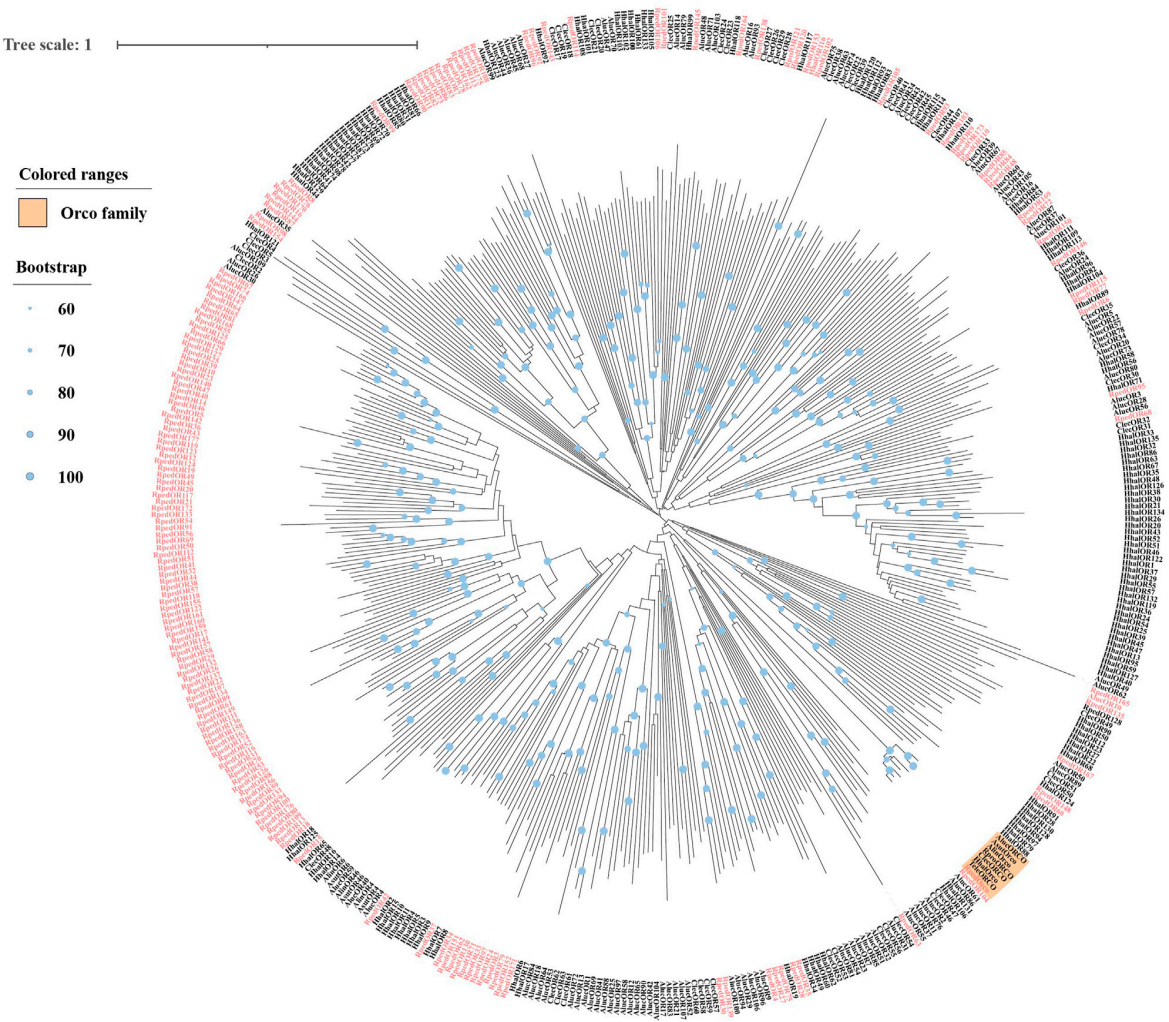
Phylogenetic relationship of ORs in *Apolygus lucorum*, *Cimex lectularius*, *Halyomorpha halys*, and *Riptortus pedestris*. The red letters represent the ORs of *R. pedestris*, and the yellow square represents the Orco family. The sequences used in this analysis are listed in Supplementary Table S4.

**FIGURE 2:**
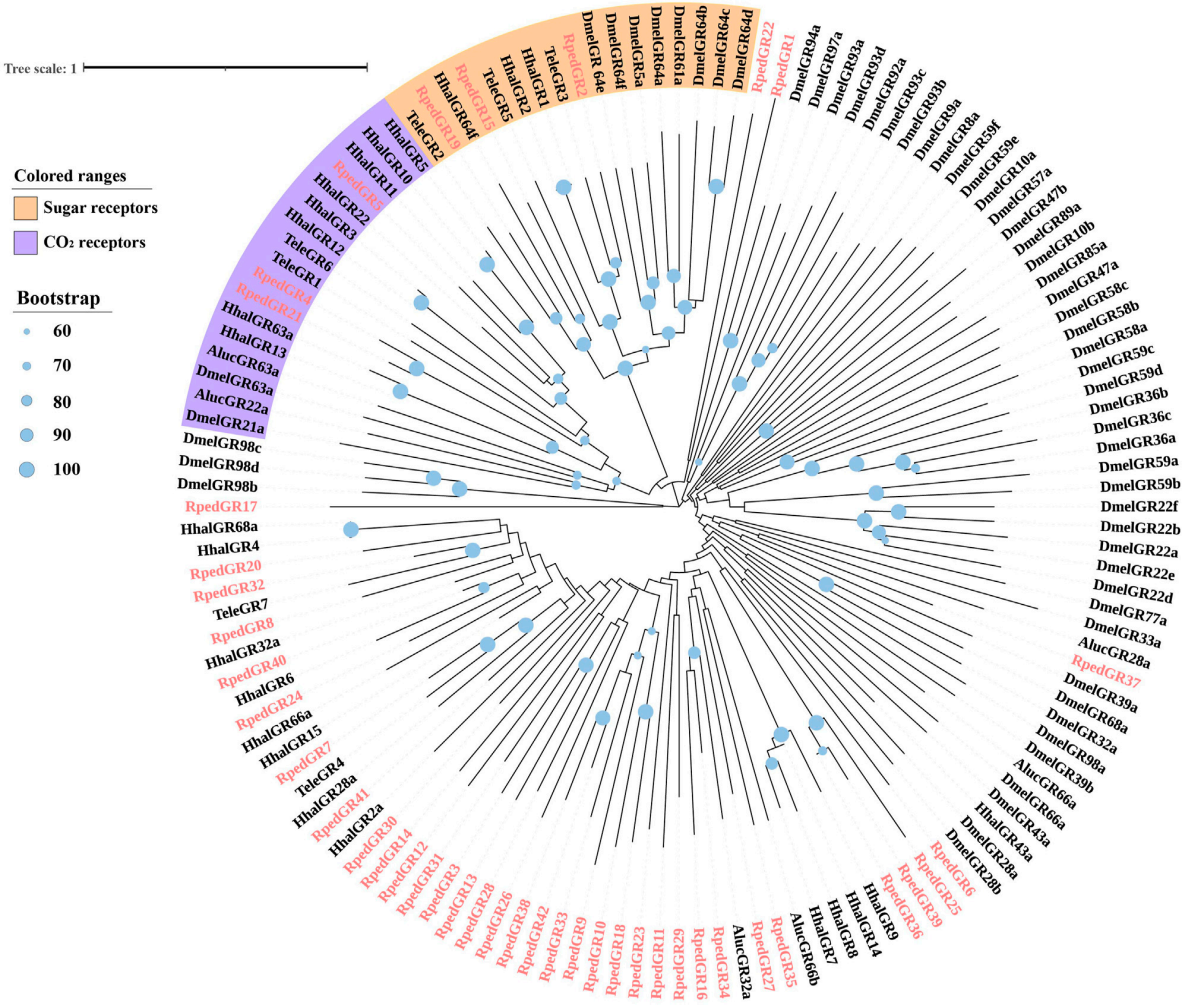
Phylogenetic relationship of GRs in *Apolygus lucorum*, *Halyomorpha halys*, *Tropidothorax elegans*, *Drosophila melanogaster* and *Riptortus pedestris*. The sequences used in this analysis are listed in Supplementary Table S4.

**FIGURE 3:**
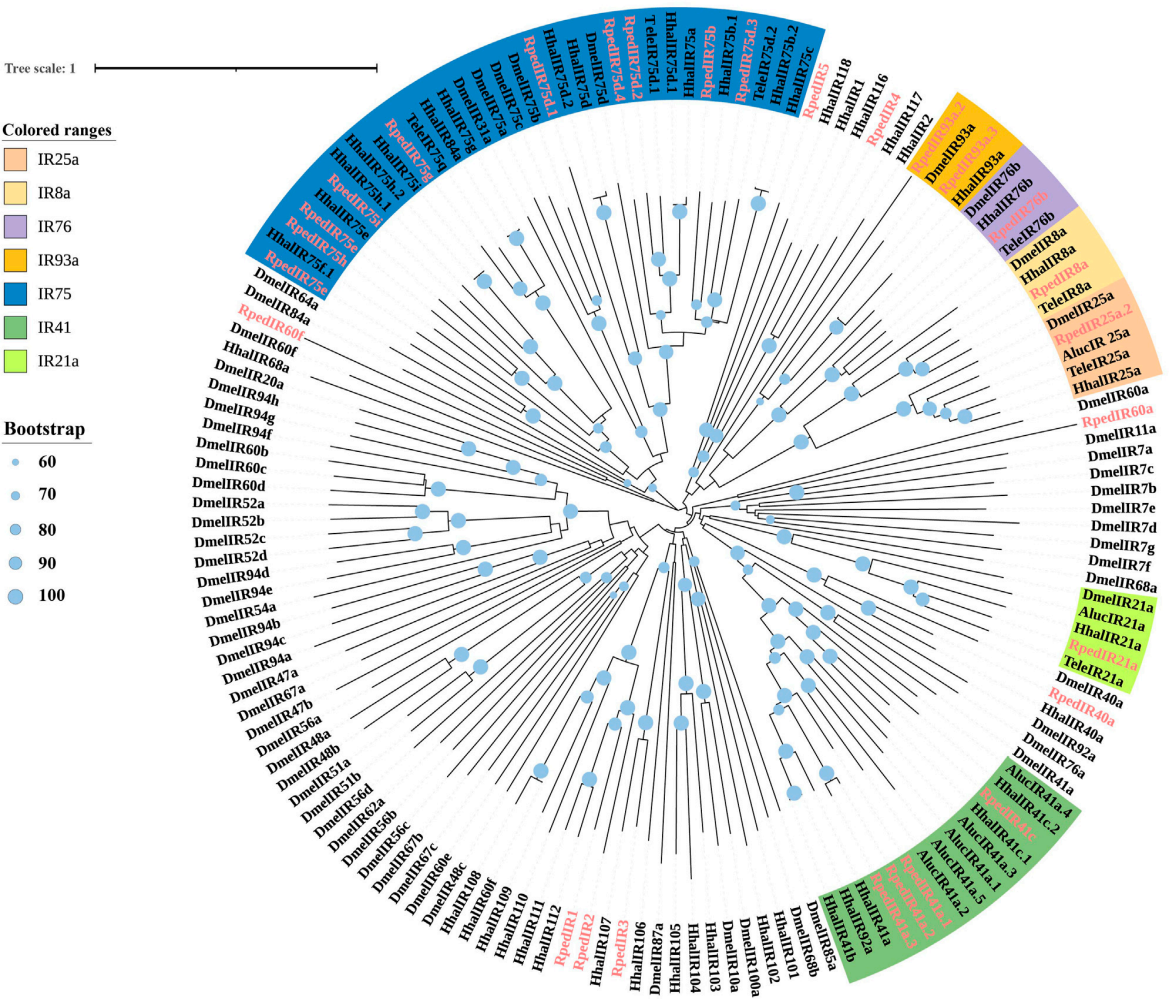
Phylogenetic relationship of IRs in *Apolygus lucorum*, *Halyomorpha halys*, *Tropidothorax elegans*, *Drosophila melanogaster* and *Riptortus pedestris*. The sequences used in this analysis are listed in Supplementary Table S4.

A phylogenetic tree was reconstructed using 469 ORs from *R. pedestris*, *Ap. lucorum*, *H. halys*, and *Cimex lectulari*, to understand the relationships ORs between *R. pedestris* and other hemipteran species. The phylogenetic relationship showed that *RpedOrco* gene was clustered in the branch with Orcos from other species with high bootstrap values. Meanwhile, other RpedORs are distributed in various branches (Figure 1). The phylogenetic tree of GRs, constructed using 136 GRs from *R. pedestris*, *Ap. lucorum*, *H. halys*, *T. elegans*, and *D. melanogaster*, showed that RpedGR4, RpedGR5, and RpedGR21 were clustered in the CO_2_ receptor subfamily, and RpedGR2, RpedGR15, and RpedGR19 were classified into the sugar receptor subfamily (Figure 2). In the IR phylogenetic tree, 151 IRs from *R. pedestris*, *Ap. lucorum*, *H. halys*, *T. elegans*, and *D. melanogaster* were divided into several branches. Among the 31 RpedIRs, four putative IR co-receptors (RpedIR8a, RpedIR25a, RpedIR76b, and RpedIR93a) were clustered on one branch. RpedIR21aand three RpedIR41a were also clustered with IR21a and IR41 subfamilies, respectively (Figure 3).

### 3.3 Genomic distribution of chemosensory receptors

**FIGURE 4:**
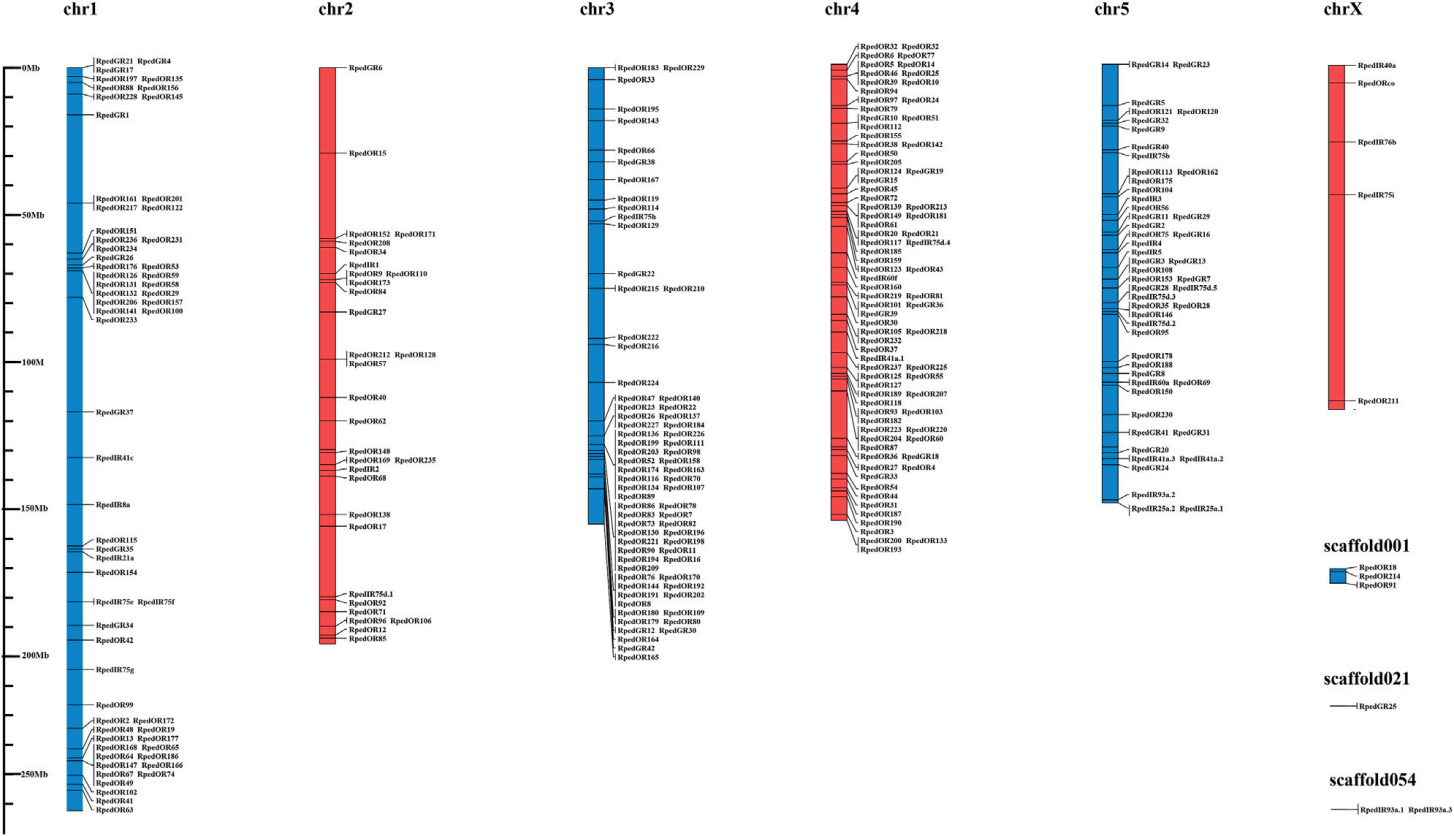
Localization of chemosensory receptors in the *Riptortus pedestris* genome.

To clarify the location of the chemosensory receptors, we located the position of 237 *RpedORs*, 42 *RpedGRs*, and 31 *RpedIRs* on the chromosomes of *R. pedestris*. The results showed that all identified chemosensory receptors were distributed on six chromosomes and three scaffolds. Among them, chr4 contained the most significant number of chemosensory receptor genes (72 ORs, 7 GRs, and three IRs), followed by chr3 (66 ORs, 5 GRs, and one IRs). The number of chemosensory receptors on chrX was the smallest, with only five genes, including *RpedOrco*. In addition, three scaffolds not spliced to the present chromosome also contained six chemosensory receptor genes (Figure 4). To better understand the gene structure of chemosensory receptors, we analyzed the introns and exons of these chemosensory receptors. The results showed that most *RpedORs* contained 4-6 exons, while 36 *RpedORs* possessed 7-9 exons. Furthermore, most *RpedGRs* contained 4-5 exons. Compared with *RpedORs* and *RpedGRs*, *RpedIRs* had significantly more exons, most of which contained over eight exons (Supplementary Table S3).

### 3.4 Expression of *RpedORs* in antennae of *R. pedestris* nymphs and adults

**FIGURE 5:**
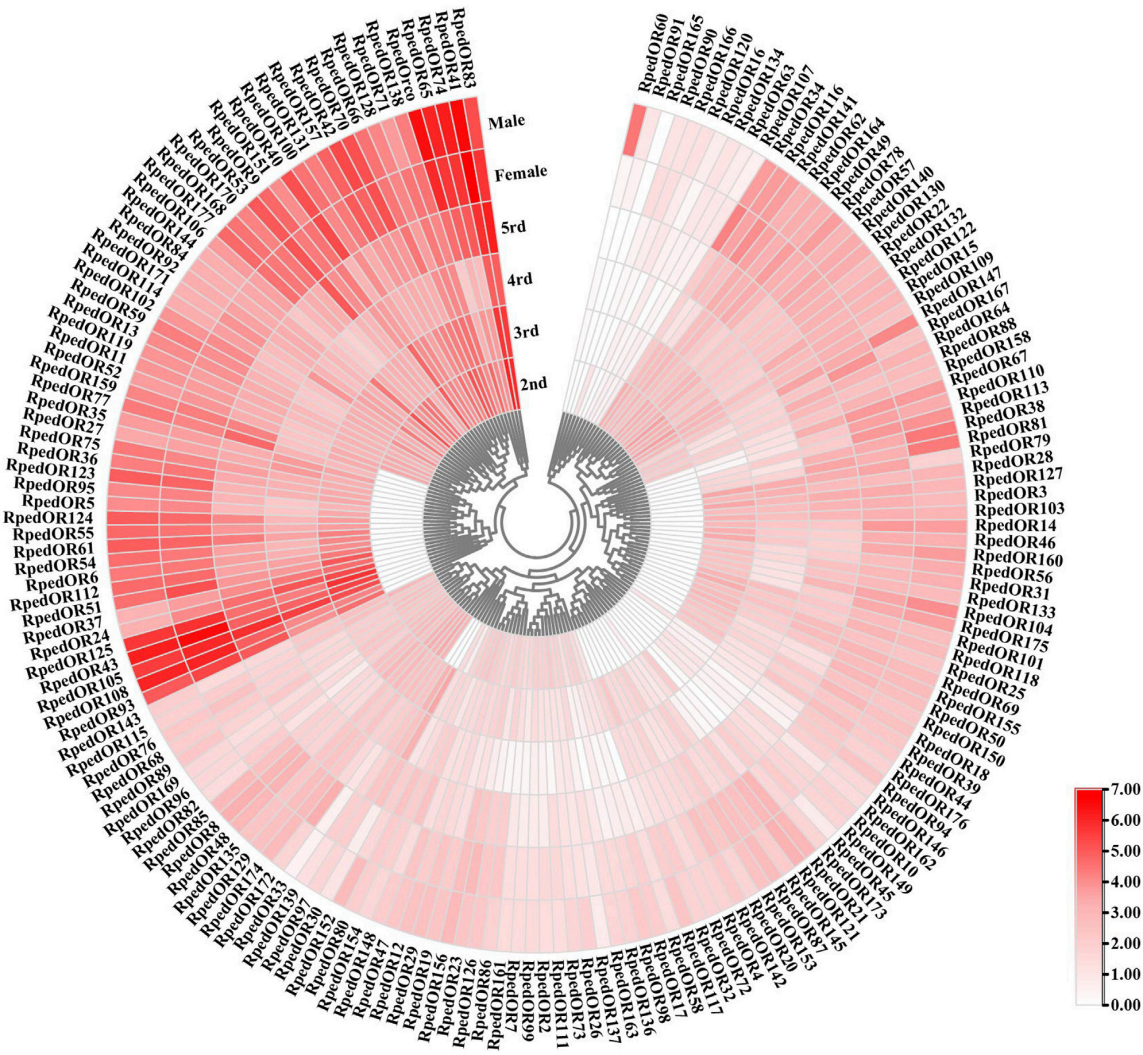
Expression profiles of olfactory receptor genes in the antennae of *Riptortus pedestris* nymphs and adults. Expression levels of the OR genes in the six transcriptomes are represented as heat plots based on log-transformed TMM values.

To better predict the role of *RpedORs* in olfactory recognition, we used RNA-seq to analyze the expression of 237 *RpedORs* in the antennae of the 2^nd^-5^th^ instar nymphs and adults (Supplementary Table S3). The heatmap showed that 177 *RpedORs* (over 300 aa) in the antennae was roughly divided into two branches based on the expression values, one highly expressed in the antennae and the other low. In the branch of high expression, the expression values of *RpedORs* increased with the development stage of the bean bugs (Figure 5). For individual genes, there was a clear bias for male and female expression. For example, the expression level of *RpedOR60* in male antennae was significantly higher than in female antennae. In contrast, *RpedOR167* is expressed higher in the antennae of females than of males (Figure 5). Interestingly, some *RpedORs* were significantly expressed in the antennae of 2^nd^- and 3^rd^-instar nymphs in comparison to the adults (e.g., *RpedOR106*).

**FIGURE 6:**
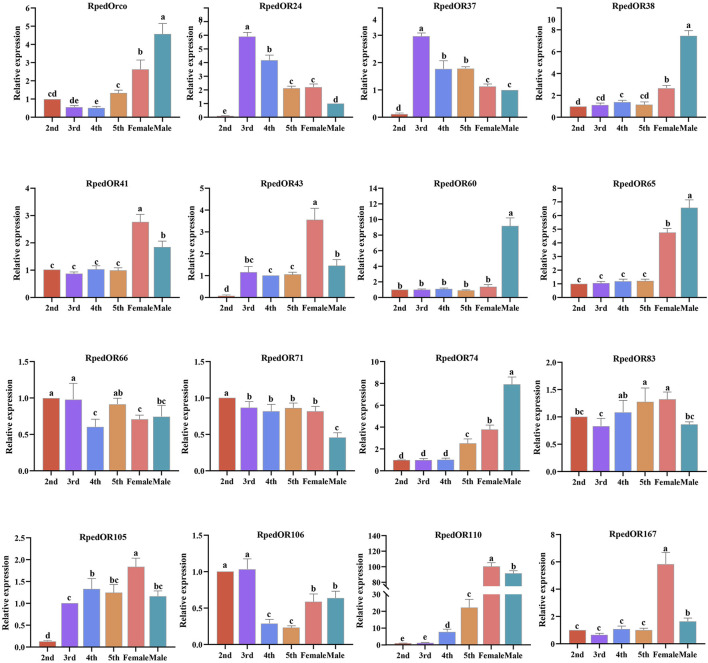
qRT-PCR based relative expression levels of several *RpedOR* genes in the antennae of nymphs, male and female *Riptortus pedestris*. Data presented are the mean of three independent biological replicates +standard error. Different letters represent significant differences according to a one-way ANOVA followed by a Tukey’s multiple comparison test (*p* < 0.05, n = 3).

In order to ensure the accuracy of transcriptome data, we selected 16 *RpedORs* with high TMM values and verified the expression of RNA-seq through qRT-PCR tests. The results showed that the expression trend of these ORs in the antennae of nymphs and adults was consistent with the results of RNA-seq (Figure 6).

### 3.5 Sequence analysis of RpedOrco

**FIGURE 7:**
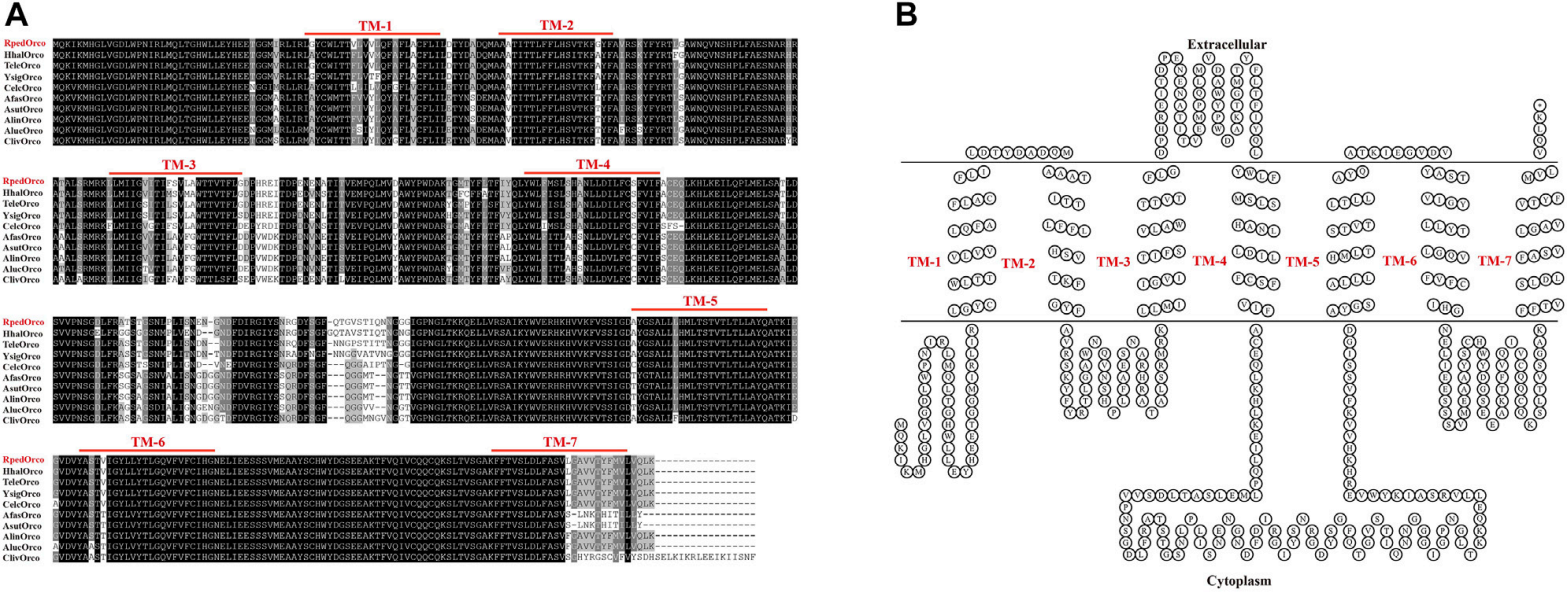
Sequence analysis of RpedOrco. **(A)** Amino acid sequence alignment of RpedOrco with other Orcos from Hemiptera insects. Afas: *Adelphocoris fasciaticollis*; Alin: *Adelphocoris lineolatus*; Asut: *Adelphocoris suturalis*; Aluc: *Apolygus lucorμm;* Cliv: *Cyrtorhinus lividipennis*; Clec: *Cimex lectularius*; Hhal: *Halyomorpha halys*; Tele: *Tropidothorax elegans*; Ysig: *Yemma signatus*. **(B)** Seven-transmembrane topology of representative RpedOrco. The double line represents the membrane region with labeled extracellular and cytoplasmic sides. TM: transmembrane. The Orco sequences used in this analysis are listed in Supplementary Table S4.

Increasing reports demonstrate that Orco receptors are highly conserved during insect evolution. Sequence alignment of RpedOrco with Orcos from other Hemiptera insects (*A. fasciaticollis*, *A. lineolatus*, *A. suturalis*, *Ap. lucorμm*, *C. lectularius*, *Cyrtorhinus lividipennis*, *H. halys*, *T. elegans*, *Y. signatus*) revealed that these Orco sequences were highly conserved. The similarities of RpedOrco with other hemipteran insects were 80.84% (AfasOrco), 83.58% (AlinOrco), 82.74% (AlucOrco), 80.84% (AsutOrco), 86.92% (ClecOrco), 77.64% (ClivOrco), 93.26% (HhalOrco), 94.94% (TeleOrco) and 93.04% (YsigOrco), respectively. Multiple amino acid sequence alignment showed that Orco was highly conserved in Hemiptera insects and had the highest degree of identity in the C-terminal sequences (TM5-TM7) (Figure 7).

### 3.6 Silencing *RpedOrco* impairs the response to aggregation pheromone

**FIGURE 8:**
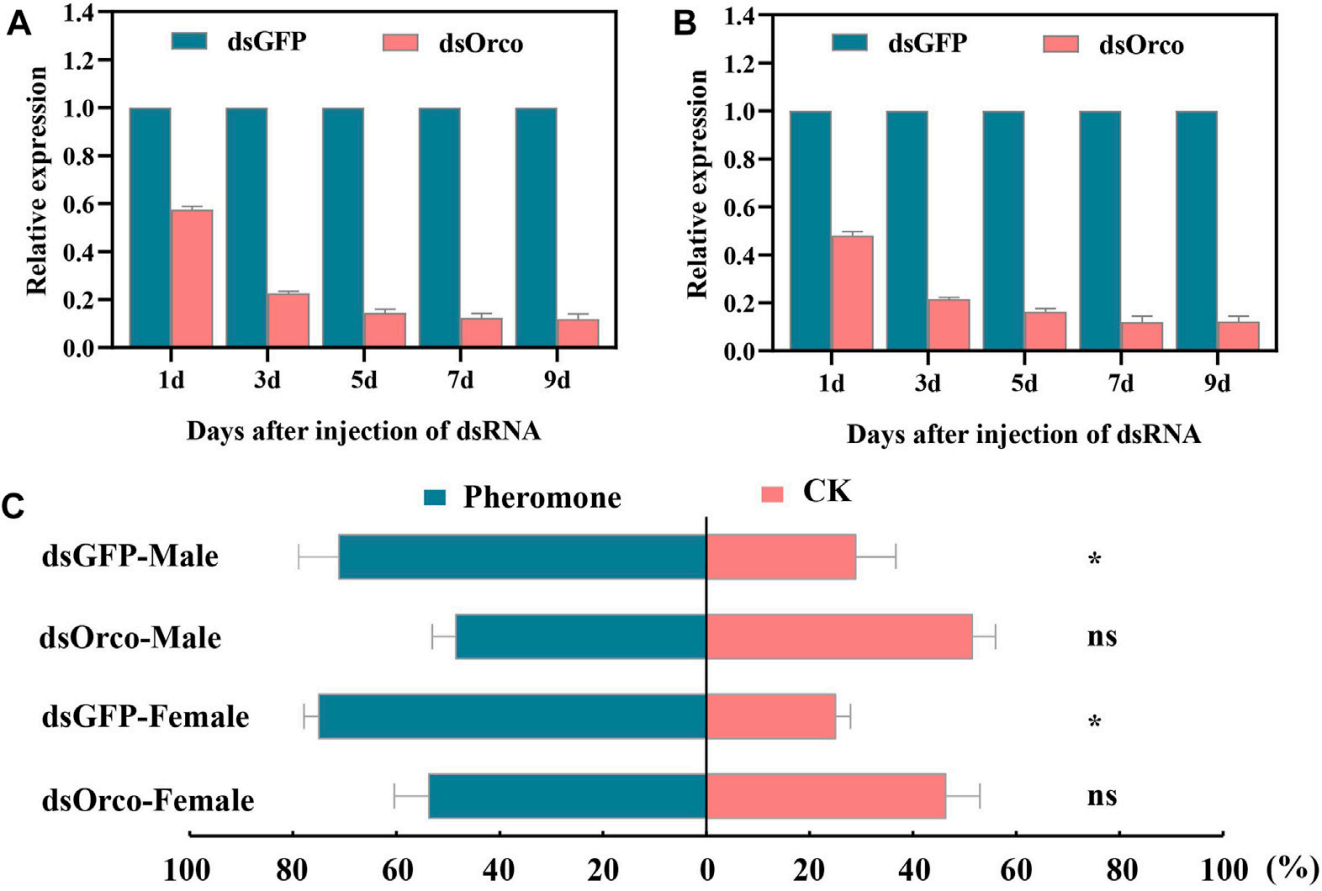
Silencing *RpedOrco* impaired the response of *Riptortus pedestris* to aggregation pheromone. **(A)** Silence efficiency of *RpedOrco* gene in male antennae after dsRNA injection; **(B)** Silence efficiency of *RpedOrco* gene in female antennae after dsRNA injection; **(C)** Behavioral response of dsOrco and dsGFP-injected *Riptortus pedestris* to aggregation pheromone lure. The asterisk represents significant difference (*p <* 0.05).

To better investigate the role of *RpedOrco* in physiology, we silenced the *RpedOrco* using RNAi technology. Few injected bugs died during our experiment, suggesting the dsRNA injection at the head of the adults was feasible. The silencing efficiency determined by qRT-PCR showed that the knockdown rate soared to approximately 80% on the third post-injection day and maintained at >85% in the following days (Figure 8). On the seventh day of post-injection, a decrease of more than 85% in *RpedOrco* expression was observed in bugs injected with dsOrco (Figure 8). Consequently, the bugs at 7 days post-injection were selected for behavioral bioassays. The results of behavior bioassays showed that more dsOrco-injected *R. pedestris* (male: *t* = 0.446, *p* = 0.669; female: *t* = 0.784, *p* = 0.477) failed to locate beans with aggregation pheromone, while most dsGFP-injected bugs succeeded (male: *t* = 3.826, *p* = 0.019; female: *t* = 12.247, *p* < 0.001) (Figure 8C).

## 4 Discussion

Insects’ behavior highly relies on their accurate chemosensory system, in which chemosensory receptors play critical roles in detecting chemical signals. Particularly, ORs have been more widely studied. Since the first discovery of insect ORs in *D. melanogaster* (Clyne et al., 1999; Gao et al., 1999), ORs have been widely studied in a variety of insects, including dipterans, hymenopterans, lepidopterans, coleopterans, and hemipterans (Fan et al., 2022; Tian et al., 2022). The present study identified 310 candidate chemoreceptors from the reported *R. pedestris* genome, including 237 RpedORs, 42 RpedGRs, and 31 RpedIRs. Furthermore, their phylogenetic relationship, localization in chromosomes, and expression profile were also analyzed according to bioinformatics data.Moreover, our results of RNAi and behavioral bioassays demonstrated that RpedOrco is essential for *R. pedestris* detecting aggregation pheromones.

Genome and transcriptome were the mainstream method for identification and exploration of chemoreceptor genes. Previously, transcriptome annotation was the exclusive channel for identifying chemoreceptors in non-model species, mainly due to the lack of their genomic information. With the progress of sequencing technology, increasing numbers of insect genome data are unveiled and available, enabling the genome-wide identification of chemosensory genes. Generally, more ORs could be identified from the genome than transcriptome data because some insect ORs are highly diverse sequences and have low expression in specific issues (Tian et al., 2022). In *Ap. lucorum*, for instance, 155 ORs could be annotated from genome data (Tian et al., 2022), while only 110 ORs were reported in the antennal transcriptome (An et al., 2016). Similar to ORs, much more *Rhynchophorus ferrugineus* GRs were identified from the genome (65) than antennal transcriptome (16) (Engsontia and Satasook, 2021). These results indicate that genome-wide identification of chemosensory genes is more feasible and reliable compared with the transcriptome-based method (Tian et al., 2022). For *R. pedestris*, we also identified significantly more chemoreceptors than the previous report by Song et al. (2017), in which 188 ORs, 6 GRs, and two IRs are annotated from the antennal transcriptome.

The number of ORs is higher diverse among insects, ranging from as few as ten in *Pediculus humanus* to more than 400 in a social ant *Camponotus foridanus* (Zube and Rossler, 2008; Kirkness et al., 2010; Fan et al., 2022). In hemipteran species, the number of ORs also varies enormously. Tian et al. (2022) reannotated 887 OR from 11 species in nine hemipteran families and found that the OR numbers vary from 9 to 13 (*Bemisia tabaci*) to 155 (*Ap. lucorum*). Based on the whole-genome data, we found *R. pedestris* has a much larger odorant reception system than other hemipterans, with identification of up to 237 RpedORs. More RpedORs seemingly betoken that *R. pedestris* would have a broad host recipe because the number of ORs is reported to associate with the host breadth in many insects (Mitchell et al., 2019). In Hemiptera, however, Tian et al. (2022) demonstrated that OR number is not directly parallel to the diversity of the host. Thus, it is reasonable that *R. pedestris* has a considerably larger number of ORs (237) than polyphagous *Aphis gossypii* (47 from the genome data), which feeds on over 700 host plants worldwide (Cao et al., 2014; Tian et al., 2022).

The diversity of OR numbers among species is attributed to gain (via tandem duplication) and loss (via pseudogenization and deletion) events, which were considered as a consequence of random genomic drift or adaption to environment (Dong et al., 2009; Mendivil and Ferrier, 2012; Andersson et al., 2019; Tian et al., 2022; Zhang et al., 2023). The discovery of some tandem replications of the ORs of both ants and bees suggested the tandem replication in Hymenoptera is the main mechanism of OR amplification (Robertson and Wanner, 2006; Zhang et al., 2023). Moreover, the OR gene family in hemipteran insects has undergone rapid expansion, with the existence of gene replication as well (Nei et al., 2008; Tian et al., 2022). For instance, *Acyrthosiphon pisum* ORs form two large lineage-specific subfamily expansions which include some tandem arrays (ApOR20-22 on SCAFFOLD42, ApOR23-24 on SCAFFOLD6001, ApOR40-41 on SCAFFOLD150003), and most of the genes in two main clades have apparently undergone relatively recent duplications of genes (Smadja et al., 2009). In the present study, we found some RpedOR genes exist as genomic clusters, mostly concentrating on chr3 and chr4 (Figure 4). This result suggested that gene duplication also existed in *R. pedestris* that led to the increase of RpedORs number.The insect OR genes are mainly expressed in the antennae and other olfactory-related accessories, where volatile semiochemcials are recognized and subsequently trigger various olfactory-driving behaviors (Leal et al., 2013; Lombardo et al., 2017). To further explore the roles of ORs in olfactory recognition, we evaluated the *RpedORs*’ expression in antennae at different development stages of *R. pedestris*. Along with the development of nymphs, the majority *RpedORs* positively expressed in antennae of an increasing instar (Figure 5), suggesting that these RpedORs may constantly play roles in olfactory recognition during *R. pedestris* development. However, a few *RpedORs* (e.g., *RpedOR30* and *RpedOR33*) are significantly expressed in the antennae of younger nymphs than adults, indicating that different *RpedORs* participate in specific olfactory responses, such as foraging or avoiding predators. In addition, many *RpedORs* showed significant expression differences between adults and nymphs (Figure 5), presumably because the adults shoulder more olfactory-related responsibilities, such as host shift, migration, finding mates, and location of optimal oviposition sites. These are the main reasons for the difference in OR expression in different insect stages (Harwood et al., 2009; Vaello al., 2017). For *R. pedestris*, additionally, nymphs and adults display conspicuous polymorphism in chemical production and emission, indicating they also smell different from conspecific volatiles (Xu et al., 2021). The identification and function of RpedORs associated with the recognition of conspecific secretions would be our further study focus.

Among all olfactory receptors, Orco is the most special and obligate one. Abundant literature has documented that insect Orcos are highly conserved among species, while specific ORs are relatively diverse with low similarity (Jones et al., 2005; Sun et al., 2020b). In this study, RpedOrco also shows high homology with Orcos from other hemipterans, with the highest similarity to HhalOrco (93.26%) in *H. halys* (Figure 7), which is consistent with previous findings in other insects (Hansen et al., 2014; Franco et al., 2016). In addition, the highly-conserved C-terminal region of Orco suggests that this region may associate with the functional interaction between ORx and Orco proteins (Butterwick et al., 2018; Zufall and Domingos, 2018; Sun et al., 2023). As an obligate unit of olfactory signal transmission in insect ORNs, Orco is predicted to assist specific ORs in recognizing chemical stimuli by forming a heteromeric ORx-Orco rather than singly responding to odorants (Stengl and Funk, 2013). Once Orco does not function properly, the insect olfactory transduction process is interrupted, impairing subsequent odorant detection. In light of its necessity in olfactory recognition, Orco attracts increasing focus as a target for developing pest control agents (Fan et al., 2022). To date, mutants of many insect species have been generated to characterize and investigate the function of *Orco* through gene silencing with RNA interference (RNAi) or gene editing with CRISPR-Cas9 (Tateishi et al., 2022; Wang et al., 2022). For example, RNAi-based silencing of the *Orco* gene in *Protaetia brevitarsis* adults significantly impaired their location of aggregation pheromones and food sources (Zhang et al., 2021). Knocking out of the *Orco* gene through CRISPR-Cas9 seriously affects the olfactory-related behaviors as well, which has been confirmed in *Anopheles coluzzii* (Sun Y. et al., 2020), *A. sinensis* (Wang et al., 2022), *Bactrocera dorsalis* (Xu et al., 2022), *B. mori* (Liu et al., 2017), *Harpegnathos saltator* (Yan et al., 2017), *Helicoverpa armigera* (Fan et al., 2022), *Locusta migratoria* (Li et al., 2016), *Spodoptera frugiperda* (Sun et al., 2023) and *S. littoralis* (Koutroumpa et al., 2016). Our results of silencing *RpedOrco* in *R. pedestris* reached the similar conclusion. The dsOrco-injected bugs, which showed a high knockdown rate of *RpedOrco*, lost the location activity to specific aggregation pheromones (Figure 8). We also tested the silencing efficiency of *RpedOrco* in nymphs, the results showed that it did not reach 85% within 3 days after injection. Based on the fact that the inter-age period lasts only about 3 days (Kim and Lim, 2010; Rahman and Lim, 2017), it cannot accurately define the instar during the behavioral bioassays. Nevertheless, the results that both RNA-seq and qRT-PCR showed the relatively high expression of *RpedOrco* in antennae of *R. pedestris* at different development stages (Figures 5, 6) also indicated its constant role in olfactory recognition.

## Data Availability

The datasets presented in this study can be found in online repositories. The names of the repository/repositories and accession number(s) can be found below: https://www.ncbi.nlm.nih.gov/sra/; SRR21820231-SRR21820236.
