# Supplementary figures and images for: Genome-wide identification of candidate chemosensory receptors in the bean bug Riptortus pedestris (Hemiptera: Alydidae) and the functional verification of its odorant receptor co-receptor (Orco) in recognizing aggregation pheromone
Source: Front Physiol. 2023 Jul 14;14:1224009. doi: 10.3389/fphys.2023.1224009 (PMC10375722; doi:10.3389/fphys.2023.1224009)

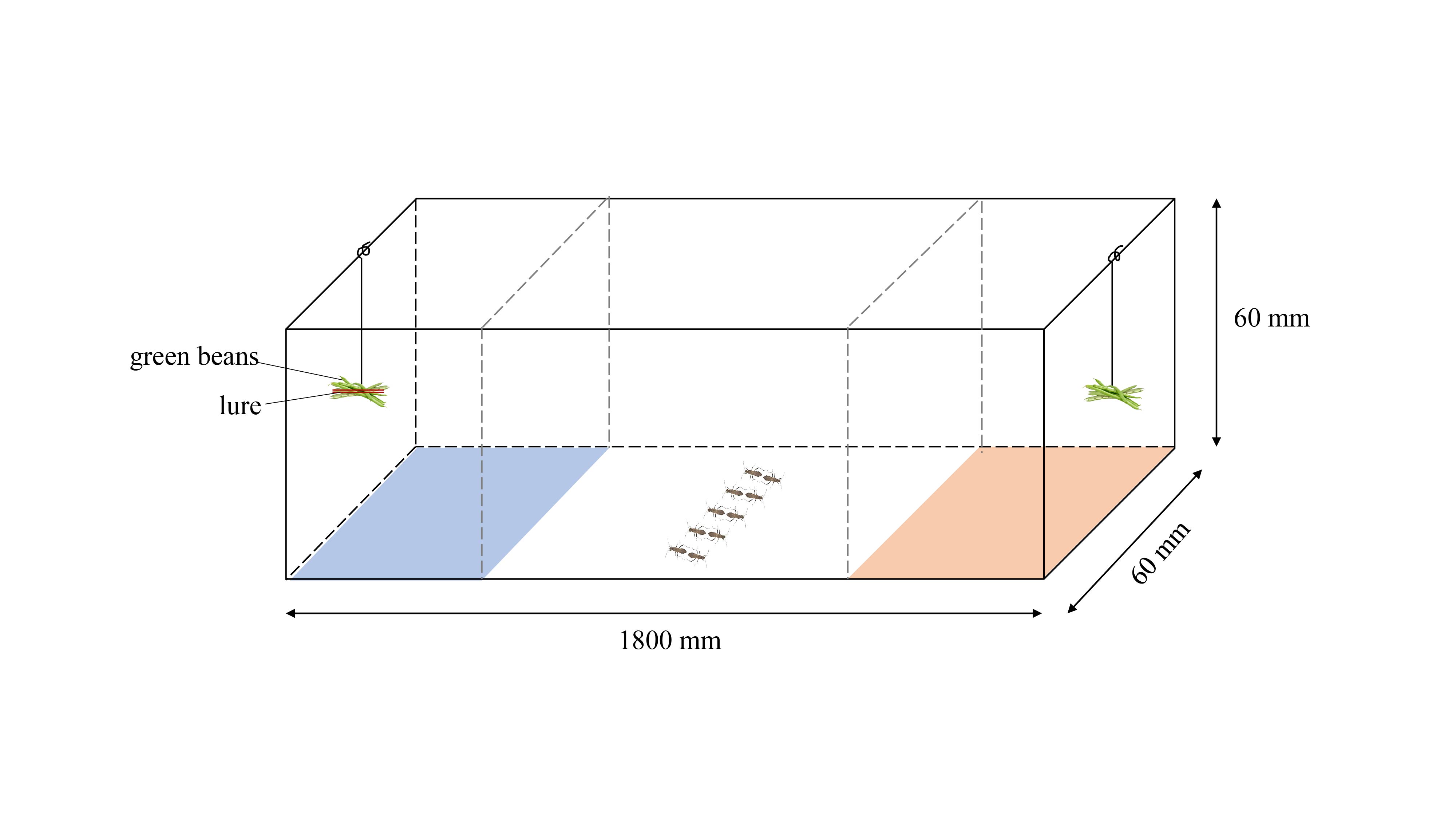

Supplement: Supplementary file 3 [file Image1.JPEG]
